# Optimization of accelerated aqueous ethanol extraction to obtain a polyphenol-rich crude extract from rambutan (*Nephelium lappaceum* L.) peel as natural antioxidant

**DOI:** 10.1038/s41598-022-25818-7

**Published:** 2022-12-07

**Authors:** Supakchon Klongdee, Utai Klinkesorn

**Affiliations:** 1 Department of Food Science and Technology, Faculty of Agro-Industry, Kasetsart University, Bangkok, 10900 Thailand; 2 Department of Food Processing and Preservation, Institute of Food Research and Product Development, Kasetsart University, Bangkok, 10900 Thailand

**Keywords:** Biophysics, Chemistry

## Abstract

An accelerated solvent extraction method was used to recover polyphenol-rich crude extract from rambutan (*Nephelium lappaceum* L.) peel, a waste product from the canning industry. The influence of extraction parameters including temperature, extraction time and ethanol concentration on extraction yield, total phenolic content, total anthocyanin content, and ABTS antioxidant activity was investigated. A Box-Behnken design and response surface methodology were used to optimize the extraction process. Optimal conditions were obtained at temperature, extraction time, and ethanol concentration of 60 °C, 34 min, and 54 vol%, respectively. These optimum conditions gave 333.01 ± 5.84 mg gallic acid/g, 318.28 ± 5.56 mg cyanidin-3-O-glucoside/g, and 3.05 ± 0.04 mmol Trolox/mg for total phenolic content, total anthocyanins content, and ABTS activity, respectively with extraction yield of 28.68 ± 1.48 wt%. Important active compounds found in the extract were geraniin, ellagic acid, shikimic acid and corilagin. Crude extract concentrations of 50–500 mg/kg retarded linoleic acid oxidation but efficacy was lower than synthetic antioxidants at 200 mg/kg. The current findings indicated that accelerated aqueous ethanol extraction was an effective method for the recovery of a crude extract rich in polyphenols from rambutan peel with the potential to be used as a natural antioxidant.

## Introduction

Rambutan is a native fruit in Southeast Asian regions, of Indonesia, Malaysia, the Philippines, Vietnam, and Thailand. The fruit is an ovoid berry and fully covered with spinterns. Rambutan peel accounts for around 50 wt% of the whole fruit weight^1^. Normally, rambutan peel is discarded as waste material from the canning industry. For Thailand, the Office of Agricultural Economics (OAE) reported that the annual production of fresh rambutan fruit from 2018 to 2020 was approximately 270 metric tons. Thus, an estimated average discard for rambutan peel is 135 metric tons annually. The recovery of valuable compounds from by-products or agro-industrial waste has recently attracted increasing interest as an economic method to resolve environmental problems. Numerous studies reported that rambutan peel extracts exhibit antioxidant, antimicrobial, anti-inflammatory, antihyperglycemic and antidiabetic activities, with high phenolic contents^2–5^. Rambutan peel extract showed potential for used as a natural antioxidant in various products including food, pharmaceuticals and cosmetics^6–8^. In terms of toxicity, the LD50 (median lethal dose) value of rambutan peel extract was more than 5000 mg/kg body weight in oral acute toxicity test that shows a very low level of toxicity^9^. Therefore, extraction of polyphenols from rambutan peel using a proper extraction process could significantly reduce the agro-industrial waste.

Various extraction techniques for recovery of polyphenols from by-products or agro-industrial wastes have been studied including maceration^10^, heat-assisted extraction^11^, ultrasound assisted extraction (UAE)^12,13^, microwave assisted extraction (MAE)^12,14^, enzyme-assisted extraction^15^, subcritical water extraction (SWE)^16^, and accelerated solvent extraction (ASE)^17,18^. Most of the mentioned extraction processes have been applied to recover the polyphenols and other bioactive compounds from rambutan peel. However, ASE method, also known as pressurized liquid extraction (PLE), enhanced solvent extraction (ESE) and high-pressure solvent extraction (HPSE) is understudied for rambutan peel extract. In this technique, elevated temperature and pressure are applied to extract the compounds from plant raw materials. Advantages of ASE include easy automation, faster sample extraction, lower solvent consumption as a reproducible, and efficient extraction method^18^.

Extraction temperature, time, and solvent are the most independent factors impacting extraction efficiency using ASE^17–20^. Herrero et al.^17^ used ASE for optimization of the extraction of antioxidants from the microalga *Spirulina platensis*. They found that the optimal temperature and time varied with the extraction solvent. The optimal conditions also depended on the plant materials. For example, the optimal temperature, time, and solvent were 80 °C, 10 min, and 64 wt% aqueous ethanol for extraction of phenolic compounds from *Passiflora* species^18^. For the extraction of polyphenol from avocado peel, the optimal extraction conditions were 200 °C as the extraction temperature and 50 vol% aqueous ethanol as the extraction solvent^19^. A process temperature of 200 °C, extraction time of 20 min, and ethanol of 77 vol% had been reported as optimal conditions for extraction of bioactive compounds from Pomegranate (*Punica granatum* L.) peel^20^.

In foods, recently, natural ingredients are currently emphasized use as a replacement for synthetic ingredients. Recovery of active compounds from natural sources to use as food ingredients is the solution for the consumer-driven trend for future food. In addition, there is a limited study that has focused on the optimization of polyphenols from rambutan peel using the ASE technique. Accordingly, the influences of process variables including extraction temperature, time and ethanol concentration for extraction of polyphenol-rich extract from rambutan peel using ASE were investigated in this work. Aqueous ethanol is considered for use as an extraction solvent in this work because of its polarity feature, which would demonstrate a high recovery, it is also safe and environmentally friendly^12,17^. The response variables were extraction yield, total phenolic content (TPC), total anthocyanin content (TAC), and ABTS activity. The phenolic profile of rambutan peel extract obtained from optimal conditions was also analyzed. The inhibition of lipid oxidation of this crude extract was also evaluated using the linoleic acid oxidation system.

## Materials and methods

### Plant material

Rambutan (*Nephelium lappaceum* L.) peel used in this research was a waste from the canning industrial supplied by Malee Group Public Company Limited (Nakhon Pathom, Thailand). Rambutan peels were washed with clean water and then sliced into small pieces. The sliced peels were dried by hot air at 70 °C until moisture content reached ~ 5 wt% and then ground to a coarse powder (5–10 mesh) and kept in a freezer (~ −18 °C) until use.

### Chemicals

Absolute ethanol and hydrochloric acid were purchased from RCI Labscan Ltd. (Bangkok, Thailand). Trolox (6-hydroxy-2,5,7,8- tetramethylchroman-2-carboxylic acid), 2,2′-azino-bis (3-ethylbenzothiazoline-6-sulfonic acid) or ABTS, Folin-Ciocalteu reagent and 2,6-di-tert-butyl-4-methylphenol (BHT) was obtained from Sigma-Aldrich (St. Louis, MO, USA). Gallic acid, sodium carbonate, potassium persulfate and potassium chloride were procured from Merck (Darmstadt, Germany). Linoleic acid was purchased from TCI: Tokyo Chemical Industry Co., Ltd. (Tokyo, Japan). Iron (II) chloride tetrahydrate was supplied from Qrec (Auckland, New Zealand). Ammonium thiocyanate was purchased from Ajax Finechem Pty Ltd. (Taren Point, NSW, Australia).

### Accelerated solvent extraction (ASE) procedure

An ASE 350 system (Dionex, Sunnyvale, CA, USA) with 100 mL stainless steel vessels was used as the pressurized solvent extractor. Approximately 15 g of dried rambutan peel was placed in an extraction cell. The extraction cells were placed in a carousel and the samples were extracted using one static cycle with 60% flush volume and 180 s purge time. The ASE parameters were set as temperature in the range from 60 to 120 °C, with aqueous ethanol concentration 50 to 90 vol% and extraction time between 10 and 50 min. The obtained extracts were evaporated to dryness using a rotary evaporator (Buchi Rotavapor R-215 Postfach Flawil, Switzerland) under 45 °C and 72 mPa until free of solvent and then stored at ~ −18 °C for further analysis.

### Conventional maceration procedure

For the conventional extraction method, the maceration procedure was performed in comparison with the ASE. Dried rambutan peel (15 g) was mixed with 54 vol% aqueous ethanol using a solvent to solid ratio of 10 mL/g. The mixture was then extract at 60 °C for 4 h with continuous stirring at 350 rpm. After that, the mixture was centrifuged at 4696 × g (Sorvall Legend X1R, Thermo Fisher Scientific, MA, USA) and then filtered to obtain the liquid fraction. The extract was evaporated until dryness and free of solvent. Finally, it was then stored at ~ -18 °C for subsequent analysis.

### Calculation of extraction yield

Amounts of rambutan peel extract from each extraction condition were measured gravimetrically and calculated as percentage of raw material used by the following equation:

1 $$Yield \left(\%\right) = \frac{Weight of dried extract}{Weight of sample} \times 100$$

### Analysis of total phenolic content

Total phenolic content (TPC) was measured using Folin-Ciocalteu reagent as previously described by Wolfe et al.^21^ with minor modifications. The crude extract was diluted with deionized water at 250 mg/L and the solution (0.2 mL) was then mixed with Folin-Ciocalteu reagent (0.2 mL) and deionized water (2.6 mL) and allowed to react for 6 min at room temperature. After that, 7% sodium carbonate solution (2 mL) was added and the mixture was kept in the dark for 90 min. The developed color was read at 750 nm using a spectrophotometer (Thermo Scientific, Madison, WI, USA). Gallic acid solution was used to construct a standard curve and the result was expressed as milligrams of gallic acid equivalent per gram of extract (mg GAE/g).

### Analysis of total anthocyanin content

Total anthocyanin content (TAC) was determined using the pH-differential method^22^. The rambutan peel extract was dissolved in deionized water to a concentration of 2 g/L. The solution was then diluted at ratio 1:1 (v/v) with two buffers, KCl buffer (0.025 M, pH 1) and sodium acetate (0.4 M, pH 4.5) and allowed to equilibrate for 15 min. The absorbance was measured at 510 nm and 700 nm using a spectrophotometer (Thermo Scientific, Madison, WI, USA) calibrated with deionized water as the blank. Total anthocyanin concentration was expressed as cyanidin-3-O-glucoside equivalent (mg C-3-G/g) and calculated using Eq. (2) as follows:

2 $$TAC (mg/g) = \frac{(A\times {M}_{W}\times DF\times 1000)}{(\varepsilon \times L\times m)}$$

where *A* is the difference in absorbance (A_510_–A_700_) between pH 1 and 4.5, *M*_*w*_ is the molecular weight of cyanidin-3-glucoside (449.2 g/mol), *DF* is the dilution factor, *ε* is the molar absorptivity coefficient (26,900 mol/L·cm), *L* is the cuvette optical pathlength (1 cm) and *m* is the weight of the sample (g).

### Analysis of antioxidant activity

Antioxidant activity of rambutan peel extract was expressed as ABTS radical scavenging capacity. The ABTS assay was modified according to the protocol described by He et al.^16^*.* The ABTS solution was prepared by dissolving 10 mg ABTS in 2.6 mL potassium persulfate (2.45 mM) and stored in the dark for 16 h at room temperature. Thereafter, ABTS working solution was obtained by diluting the stock solution in ethanol to get an absorbance of 0.700 ± 0.02 at 734 nm before usage. Samples or standard solution (1 mL) were combined with ABTS working solution (3 mL) and kept in the dark for 5 min. Absorbance was measured at 734 nm and the radical scavenging activity determined by ABTS was expressed as Trolox equivalent antioxidant capacity (mmol Trolox/mg).

### Identification of phenolic compounds

Phenolic compounds in rambutan peel crude extract obtained under optimum conditions were identified by liquid chromatography coupled with electrospray-ionization triple quadrupole time-of-flight mass spectrometry (LC-ESI-QTOF/MS). Separation was carried out in an Agilent ZORBAX SB-C18 column (4.6 × 250 mm, 3.5 μm) at 35 °C with mobile phase A 3% acetic acid–water and mobile phase B acetonitrile (0–5 min 3% B, 5–20 min 9% B, 20–35 min 16% B, 35–38 min 33% B, 38–45 min 90% B and 45–50 min 3% B). Mass spectrometry analysis was operated using a microTOF-Q II mass spectrometer (Bruker Daltonics, Bremen, Germany) with electrospray ionization (ESI), negative ion mode and mass acquisition range of 50–2000 m/z.

### Inhibition of lipid oxidation in linoleic acid system

The antioxidant activity of rambutan peel crude extract was determined by measuring the inhibition of oxidation in a linoleic acid system by the ferric thiocyanate method (FTC) following the method of Rattaya et al.^23^ and, Mulyani and Harsojuwono^24^ with some modifications. The extracts were diluted with absolute ethanol to obtain concentrations of 50, 100, 200 and 500 mg/kg. About 0.1 mL of the diluted extracts were mixed with 2 mL of 0.1 M phosphate buffer solution (pH 7.0), 1 mL linoleic acid (2.5% in ethanol 96%) and 1 mL deionized water. The mixed solution was incubated at room temperature in tightly closed glass bottles wrapped in aluminum foil and sampled every 24 h during incubation to determine the level of oxidation. The FTC method was performed by mixing 0.1 mL mixed solution, 9.7 mL of 75% ethanol, 0.1 mL of 30% ammonium thiocyanate and 0.1 mL 0.02 M ferrous chloride in 3.5% HCl. After 3 min, absorbance of the solution was measured at 500 nm. Butylated hydroxytoluene (BHT) at 200 mg/kg was used as a positive control, while absolute ethanol was used as a negative control.

### Experimental design and statistical analysis

The experiment was conducted with three independent variables, namely extraction temperature (X_1_, °C), extraction time (X_2_, min) and ethanol concentration (X_3_, %) at three levels using Box-Behnken design (BBD). The complete design consisted of 15 experimental points including three replications of the central point. The coded and actual values of the factors for the experimental designs are given in Table 1.

**Table 1:** Box–Behnken design for independent variables and observed responses.

Run	Temperature (°C)	Time (min)	Ethanol (%)	Yield (Y_1_, %)	TPC (Y_2_, mg GAE/g)	TAC (Y_3_, mg C-3-G/g)	ABTS (Y_4_, mmol Trolox/ mg)
1	−1 (60)	−1 (10)	0 (70)	16.10 ± 5.14	291.76 ± 7.52	212.37 ± 5.13	2.59 ± 0.19
2	1 (120)	−1 (10)	0 (70)	37.80 ± 1.48	246.69 ± 4.37	177.22 ± 4.32	2.34 ± 0.15
3	−1 (60)	1 (50)	0 (70)	23.90 ± 0.80	285.66 ± 8.88	257.71 ± 3.16	2.55 ± 0.07
4	1 (120)	1 (50)	0 (70)	40.00 ± 6.60	265.24 ± 4.03	130.02 ± 3.81	2.53 ± 0.26
5	−1 (60)	0 (30)	−1 (50)	28.57 ± 6.74	321.73 ± 6.84	328.77 ± 3.36	2.91 ± 0.04
6	1 (120)	0 (30)	−1 (50)	60.43 ± 9.38	167.74 ± 9.55	93.84 ± 1.67	1.69 ± 0.13
7	−1 (60)	0 (30)	1 (90)	15.23 ± 3.25	184.86 ± 9.41	133.74 ± 2.89	1.89 ± 0.27
8	1 (120)	0 (30)	1 (90)	15.47 ± 2.83	273.90 ± 9.16	74.96 ± 2.14	2.97 ± 0.07
9	0 (90)	−1 (10)	−1 (50)	40.57 ± 4.20	244.42 ± 10.32	156.26 ± 1.57	2.23 ± 0.05
10	0 (90)	1 (50)	−1 (50)	43.47 ± 8.67	250.73 ± 11.83	194.30 ± 5.50	2.35 ± 0.32
11	0 (90)	−1 (10)	1 (90)	19.83 ± 4.19	184.01 ± 10.45	88.72 ± 1.53	1.94 ± 0.17
12	0 (90)	1 (50)	1 (90)	29.07 ± 4.05	220.16 ± 7.72	68.44 ± 7.91	2.34 ± 0.19
13	0 (90)	0 (30)	0 (70)	35.30 ± 0.24	259.75 ± 6.39	187.84 ± 2.98	2.47 ± 0.04
14	0 (90)	0 (30)	0 (70)	27.23 ± 3.82	297.52 ± 5.82	232.79 ± 4.71	2.85 ± 0.09
15	0 (90)	0 (30)	0 (70)	32.03 ± 0.99	324.84 ± 4.43	213.66 ± 3.31	2.98 ± 0.27

*TPC* total phenolic content, *mg GAE/g* milligram gallic acid equivalents per gram extract, *TAC* total anthocyanin content, *mg C-3-G/g* milligram cyanidin-3-O-glucoside per gram extract; *ABTS* 2,2′-azino-bis-3-ethylbenzothiazoline-6-sulphonic acid, *mmol Trolox/mg* millimole Trolox equivalents per milligram extract.

Experimental data from the BBD were analyzed by multiple regression to fit with the following second-order polynomial model:

3 $${ }Y = A_{0} { + }\mathop \sum \limits_{i = 1}^{3} A_{i} X_{i} + \mathop \sum \limits_{i = 1}^{3} A_{ii} X_{i}^{2} + \mathop \sum \limits_{i = 1}^{2} \mathop \sum \limits_{j = i + 1}^{3} A_{ij} X_{i} X_{j}$$

where *Y* represents the response function including extraction yield (*Y*_*1*_), total phenolic content (*Y*_*2*_), total anthocyanin content (*Y*_*3*_) and antioxidant activity (*Y*_*4*_); *A*_*0*_ is a constant; *A*_*i*_*, A*_*ii*_ and *A*_*ij*_ are the coefficients of the linear, quadratic and interactive terms, respectively and *X*_*i*_ and *X*_*j*_ represent the independent variables^25^. An analysis of variance (ANOVA) was performed to determine the lack of fit, determination coefficient (R^2^) and the effect of linear, quadradic and interaction terms on each response variable. Response surface methodology (RSM) was used to generate three-dimensional response surface plots. The experimental design, analysis of experimental data, model fitting and optimizing process were carried out using Minitab Statistical Software Cloud-Based Version (Minitab LLC., State College, PA, USA). To evaluate the differences between mean and between predicted value and observation value (one sample t-test), IBM SPSS Statistics (Thaisoftup Co., Ltd., Bangkok, Thailand) was used with statistical significance level of *p* < 0.05. Each experiment was conducted at least twice.

### Ethics approval

No approval of research ethics committees was required to accomplish the goals of this study because no experiments were involved on humans or animals, or field studies on plants.

## Results and discussion

### Effect of independent factors on response variables

The effect of extraction conditions using the BBD design on extraction yield (*Y*_*1*_), total phenolic content (TPC, *Y*_*2*_), total anthocyanin content (TAC, *Y*_*3*_) and antioxidant activity (ABTS, *Y*_*4*_) are presented in Table 1. Values of extraction yield, TPC, TAC and ABTS scavenging activity ranging from 15.2 to 60.4 wt%, 167–325 mg GAE/g, 68–329 mg cyanidin-3-O-glucoside/g, and 1.69–2.98 mmol Trolox/mg, respectively. These findings concurred with Palanisamy et al.^3^, Samuagam et al.^26^, and Yunusa^27^ who reported extraction yield and TPC at 22–30 wt% and 244–397 mg GAE/g respectively, while other studies found higher recovery of TPC (700 mg GAE/g)^28,29^, and greater antioxidant activity by ABTS assay (15.6 mmol Trolox/mg)^30^. Maran et al.^13^ found TAC to be 10.26 ± 0.39 mg cyanidin-3-glucoside/g and relatively lower than our value. Recovery effectiveness of bioactive compounds from rambutan peel depends on cultivar variety, growing location, extraction process, solvent type and operational circumstances.

### Fitting the models

**Figure 1:**
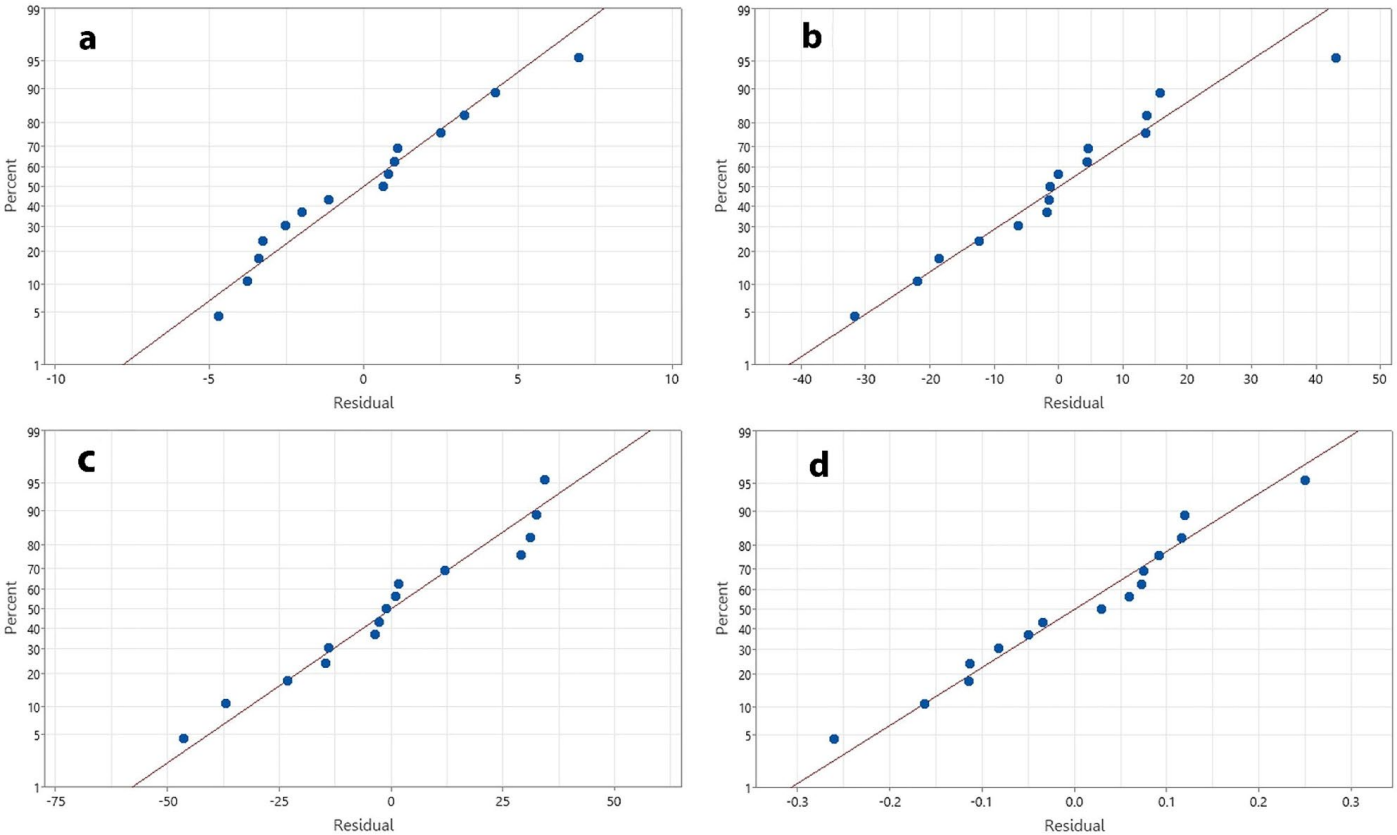
Normal probability plots of residuals for the extraction yield (**a**), total phenolic content (**b**), total anthocyanin content (**c**), and ABTS scavenging activity (**d**).

Multiple regression analysis was applied based on the experimental data (Table 1). Regression coefficients (*A*) of the independent variables, lack of fit and coefficient of determination (R^2^) of the backward second-order polynomial regression models are shown in Table 2. The adequacy and predictability of the models were considered according to the determination coefficient (R^2^), adjusted determination coefficient (R^2^_adj_), the lack of fit. All models showed high significance with *p*-values between 0.000 and 0.002. A good fit of the models with the experimental data was observed, with R^2^ and R^2^_adj_ higher than 0.86 and 0.80, respectively. These values suggested that the models could interpret more than 80% of the variation in the response variables. To support the adequacy and how well each model fitted the data, a lack-of-fit test was conducted. The *p* values of the lack of fit of the regression models were 0.609, 0.920, 0.392 and 0.922 for extraction yield, TPC, TAC and ABTS scavenging of the rambutan peel crude extract, respectively (Table 2). These values suggested that the “lack of fit” had no statistical significance (*p* > 0.05) thus, verifying the reliability of the models^31^. This suggestion was consistent with the normal probability plots (Fig. 1) indicating that for the residuals, the difference between the actual and predicted values, followed a normal distribution and formed an approximately straight line. This confirmed the satisfactory fit of the regression models to the experimental data.

**Table 2:** Regression coefficients (A) of the independent variables, coefficient of determination (R^2^) and lack of fit of the backward second-order polynomial regression models.

Source^a^	Extraction yield (%)		TPC (mg GAE/g)		TAC (mg C-3-G/g)		ABTS (mmol Trolox/mg)	
	Coefficient	*p* Value^b^	Coefficient	*p* Value^b^	Coefficient	*p* Value^b^	Coefficient	*p* Value^b^
Model		0.000		0.000		0.000		0.002
Constant (A_0_)	−41.500		401		287		4.16	
X_1_ (A_1_)	1.214	0.000	−7.63	0.056	−7.04	0.000	−0.0684	0.439
X_2_ (A_2_)	−0.1383	0.077					0.0346	0.218
X_3_ (A_3_)	0.602	0.000	7.87	0.072	11.60	0.001	0.033	0.924
X_1_X_1_ (A_11_)								
X_2_X_2_ (A_22_)							−0.000508	0.056
X_3_X_3_ (A_33_)			−0.1267	0.001	−0.1482	0.003	−0.000851	0.006
X_1_X_2_ (A_12_)								
X_1_X_3_ (A_13_)	−0.1318	0.003	0.1013	0.000	0.0734	0.014	0.000954	0.000
X_2_X_3_ (A_23_)								
R^2^	0.927		0.861		0.886		0.888	
Adjusted R^2^	0.898		0.806		0.841		0.804	
Lack of fit		0.609		0.920		0.392		0.922

^a^X_1_, X_2_ and X_3_ represented the temperature (°C), extraction time (min) and ethanol concentration (% v/v), respectively. ^b^*p* value more than 0.05 is not significantly different at 5% level. *TPC* total phenolic content, *mg GAE/g* milligram gallic acid equivalents per gram extract, *TAC* total anthocyanin content; *mg C-3-G/g* milligram cyanidin-3-O-glucoside per gram extract, *ABTS* 2,2′-azino-bis-3-ethylbenzothiazoline-6-sulphonic acid, *mmol Trolox/mg* millimole Trolox equivalents per milligram extract.

### Response surface plots

**Figure 2:**
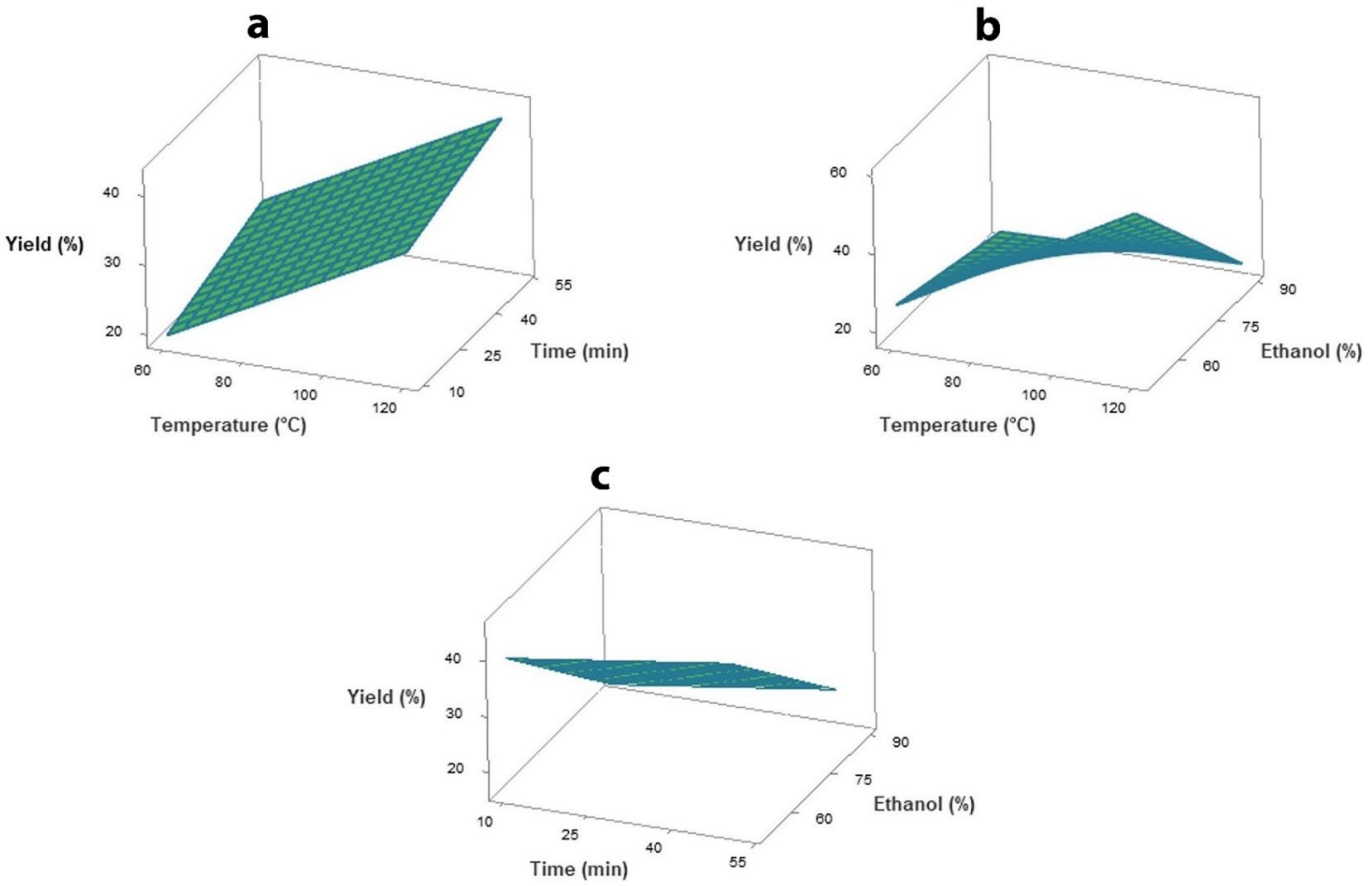
Response surface plots as a function of independent variables on the extraction yield. (**a**) temperature and extraction time, (**b**) temperature and ethanol concentration, and (**c**) extraction time and ethanol concentration.

Surface plots were used to analyze the interaction effects of the independent variables on the response variables based on the regression equation. Thus, adaptable regression models (Table 3) were used for response surface analysis. Variation of the response variables including extraction yield, TPC, TAC and ABTS scavenging activity with temperature (*X*_*1*_), extraction time (*X*_*2*_) and ethanol concentration (*X*_*3*_) were illustrated. Yield of crude extract was significantly (*p* ≤ 0.05) affected by extraction temperature and ethanol concentration (Table 2). The surface plot between extraction temperature and ethanol concentration revealed that an increase in temperature increased the yield, whereas an increase in ethanol concentration decreased the yield of extract (Fig. 2). At high temperature, molecular movement and mass transfer increased, which promoted extraction efficiency. At low ethanol concentration, water caused swelling of the rambutan peel matrix. This increased the contact surface area, thus increasing extraction efficiency^32^.

**Table 3:** Regression models as a function of independent variables for the response variables of the rambutan peel extract in terms of actual levels.

Response variables	Regression model
Extraction yield (%)	Y_Yield_ = -41.5 + 1.214X_1_–0.1383X_2_ + 0.602X_3_–0.01318 X_1_X_3_
TPC (mg GAE/g)	Y_TPC_ = 401–7.63X_1_ + 7.87X_3_–0.1267X_3_^2^ + 0.1013X_1_X_3_
TAC (mg C-3-G/g)	Y_TAC_ = 287–7.04X_1_ + 11.60X_3_–0.1482X_3_^2^ + 0.0734 X_1_X_3_
ABTS (mmol Trolox/mg)	Y_ABTS_ = 4.16–0.0684X_1_ + 0.0346 X_2_ + 0.033X_3_ –0.00051X_2_^2^–0.00085X_3_^2^ + 0.00095 X_1_X_3_

X_1_, X_2_ and X_3_ represented the temperature (°C), extraction time (min) and ethanol concentration (% v/v), respectively. *TPC* total phenolic content, *mg GAE/g* milligram gallic acid equivalents per gram extract, *TAC* total anthocyanin content, *mg C-3-G/g* milligram cyanidin-3-O-glucoside per gram extract, *ABTS* 2,2′-azino-bis-3-ethylbenzothiazoline-6-sulphonic acid; *mmol Trolox/mg* millimole Trolox equivalents per milligram extract.

**Figure 3:**
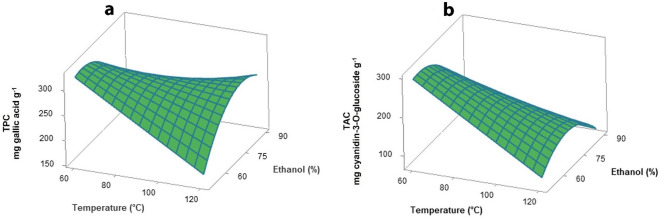
Response surface plots as a function of temperature and ethanol concentration on the total phenolic content (**a**), and total anthocyanin content (**b**).

Multiple regression analysis revealed that total phenolic content (TPC) was significantly (*p* ≤ 0.05) affected by ethanol concentration and the interaction term between temperature and ethanol concentration (Table 2). The response surface plot (Fig. 3a) shows the effect of temperature and ethanol concentration on TPC. Increase in temperature was attributed to decrease in TPC through degradation and fragmentation of phenolic compounds. Rajha et al.^11^ showed that temperature limits of each extraction differed among extraction studies, with suitable extraction temperature for phenolic compounds not more than 60–70 °C. However, some studies reported that high temperature improved the efficiency of TPC extraction. The TPC recovery of grape skins was highest (60.7 mg GAE/g) at 150 °C by the high-pressure high-temperature technique^33^. The effect of ethanol concentration was represented as a parabolic curve. The TPC increased with increasing concentration of ethanol up to 70 vol%. After that, TPC declined with increasing ethanol concentration. Water is a strong polar solvent and phenolic compounds are also polar, therefore phenolic content increased with added water according to the ‘like dissolves like’ principle^14^. At high concentrations of ethanol, solvent polarity decreased. Ethanol denatured the proteins, thereby preventing the dissolution of polyphenols and influencing the extraction rate. Extraction temperature and ethanol concentration impacted total anthocyanin content (TAC) in the same way as TPC. Lower TAC was observed with increase of temperature and ethanol concentration over 60 vol% (Fig. 3b). No significant effect of the extraction time was observed on the TPC and TAC that similar to the previous work^34^.

**Figure 4:**
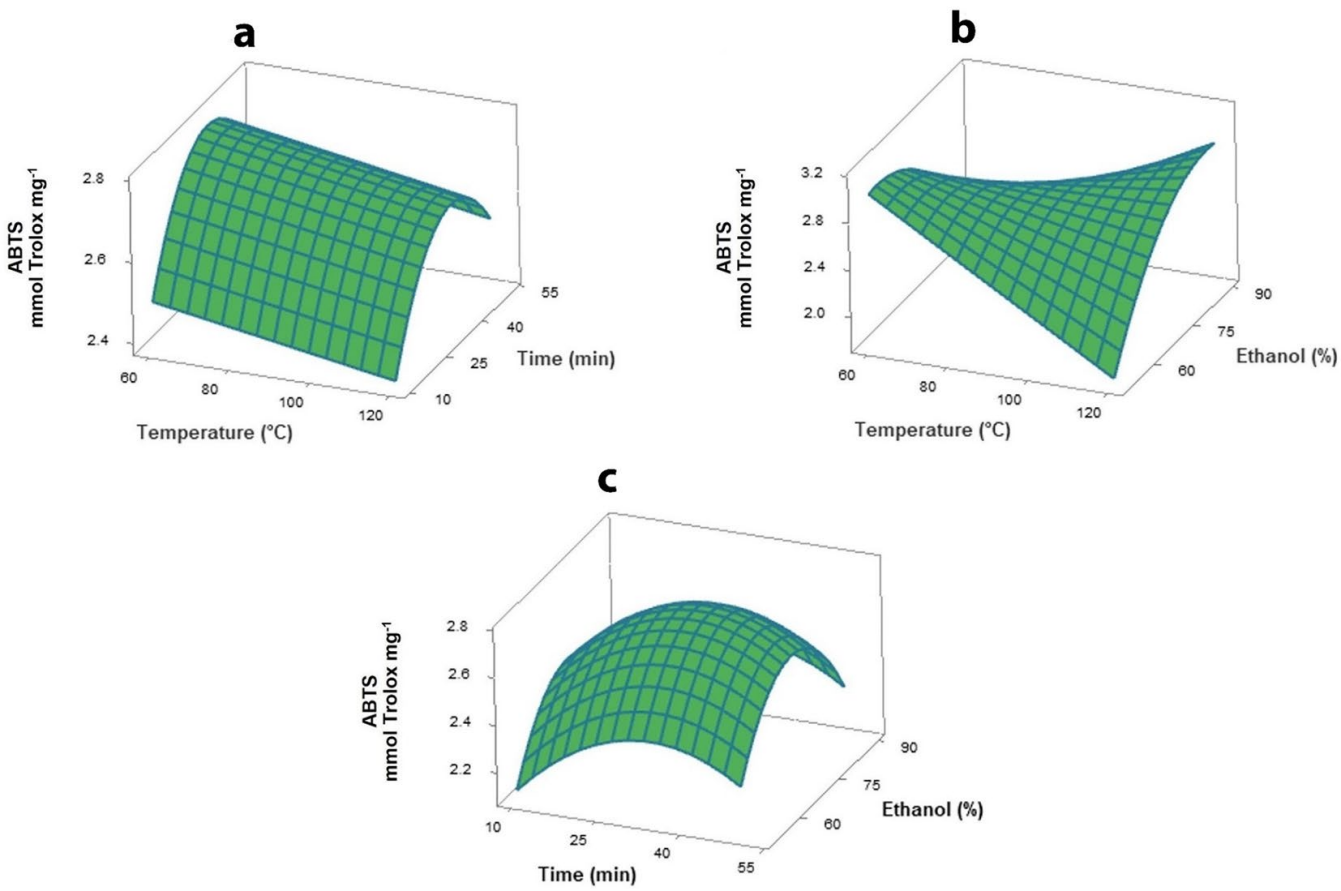
Response surface plots as a function of independent variables on the ABTS scavenging activity. (**a**) temperature and extraction time, (**b**) temperature and ethanol concentration, and (**c**) extraction time and ethanol concentration.

Antioxidant activity evaluated by ABTS assay was significantly (*p* ≤ 0.05) influenced by ethanol concentration and extraction temperature. The ABTS value increased at ethanol concentrations raised from 50 to 70 vol% and then decreased at higher concentrations (Fig. 4). The effect of ethanol concentration on ABTS was similar to TPC and anthocyanin content patterns due to the significantly (*p* ≤ 0.05) positive correlation between these phenolics and ABTS. An interactive effect of temperature and ethanol concentration was observed in the surface plot (Fig. 4b). At low ethanol concentration, increase in temperature led to decrease in ABTS value due to the destruction or degradation of some bioactive compounds at higher temperature. By contrast, at higher ethanol concentrations, the ABTS value increased, while temperature also increased, caused by facilitating the extraction of compounds with lower polarity by organic solvent and high temperature^35^.

### Process optimization and model validation

Optimizing all response variables to provide highest amounts of extracts together with maximum antioxidants was difficult because highest extraction yield was obtained at the highest temperature, which caused degradation of TPC and TAC^18^. These results were confirmed by Pearson’s correlation coefficient calculated for the response variables (Table 4), which indicated that extraction yield was not significantly correlated with the other variables. Therefore, optimal conditions for ASE of rambutan peel extract providing maximum total phenolic and total anthocyanin contents, and highest ABTS activity were determined using the RSM response optimizer. Results gave extraction temperature 60 °C, ethanol concentration 54.04 vol% and extraction time 34.02 min. Optimal production conditions were modified to 60 °C, 54 vol% and 34 min. Under these conditions, predicted TPC, TAC and ABTS activity were 326.93 mg GAE/g, 296.66 mg cyanidin-3-O-glucoside/g, and 3.04 mmol Trolox/mg, respectively.

**Table 4:** Pearson’s correlation coefficient between response variables.

Variables		Yield	TPC	TAC	ABTS
Yield	Correlation	1.000	−0.238	−0.076	−0.344
	Sig. (2-tailed)		0.393	0.079	0.210
TPC	Correlation	−0.238	1.000	0.737**	0.942**
	Sig. (2-tailed)	0.393		0.002	0.000
TAC	Correlation	−0.076	0.737**	1.000	0.525*
	Sig. (2-tailed)	0.079	0.002		0.044
ABTS	Correlation	−0.344	0.942**	0.525*	1.000
	Sig. (2-tailed)	0.210	0.000	0.044	

*Correlation is significant at the level of *p* < 0.05; **Correlation is significant at the level of *p* < 0.01; TPC: total phenolic content; TAC: total anthocyanin content; ABTS: 2,2′-azino-bis-3-ethylbenzothiazoline-6-sulphonic acid.

To evaluate model validity, ASE of rambutan peel extract was performed under optimal conditions. Average values of TPC, TAC and ABTS activity were 333.01 ± 5.84 mg GAE/g, 318.28 ± 5.56 mg cyanidin-3-O-glucoside/g and 3.05 ± 0.040 mmol Trolox/mg, respectively (Table 5). The experimental values closely matched the predicted values with insignificant differences (*p* > 0.05) and low relative errors ranging between 0.41 and 7.29%. Using the recommended optimal conditions, the experimental value of extraction yield was 28.68 ± 1.48 wt%, which was not significantly different (*p* > 0.05) from the predicted value (25.85 wt%). These results indicated good reliability and suitability of all response variables of the experimental models for accelerated aqueous ethanol extraction of polyphenol-rich crude extract from rambutan peel.

**Table 5:** Experimental and predicted values of the response variables at optimal extraction conditions of ASE along with the experimental value from conventional maceration method.

Response variables	Accelerated aqueous ethanol extraction method (ASE)			Conventional maceration method
	Predicted value	Experimental value^ns^	Relative error (%)	
TPC(mg GAE/g)	326.93	333.01 ± 5.84^a^	1.86	255.33 ± 9.98^b^
TAC (mg C-3-G/g)	296.66	318.28 ± 5.56^a^	7.29	287.27 ± 17.70^b^
ABTS (mmol Trolox/mg)	3.04	3.05 ± 0.04^a^	0.41	2.11 ± 0.07^b^

*TPC* total phenolic content, *mg GAE/g* milligram gallic acid equivalents per gram extract, *TAC* total anthocyanin content, *mg C-3-G/g* milligram cyanidin-3-O-glucoside per gram extract. *ABTS* 2,2′-azino-bis-3-ethylbenzothiazoline-6-sulphonic acid; *mmol Trolox/mg* millimole Trolox equivalents per milligram extract. ^ns^ is representation a nonsignificant difference between experimental and predicted values (*p* > 0.05, One-Sample T-Test). ^a,b^Values with different superscript letters between experimental values of ASE and conventional methods are representation a significant difference (*p* ≤ 0.05, One-Sample T-Test).

Comparing the optimal ASE with the conventional maceration performed with the similar extraction parameters except for the extraction time was conducted. It found that all response variables, including, TPC, TAC, and ABTS activity of crude extract collected from ASE, were significantly higher than that obtained from the conventional maceration method (Table 5). In addition, the extraction yield obtained from ASE method which of 28.68 ± 1.48 wt% was also significantly higher (*p* ≤ 0.05) than value obtained from conventional maceration method (21.34 ± 1.63 wt%). These results indicate the preferable application of ASE for recovery of crude extract from rambutan peel with a higher yield, enriched in polyphenols, and exceeded in antioxidant activity.

### Identification of phenolic compounds in rambutan peel crude extract

**Figure 5:**
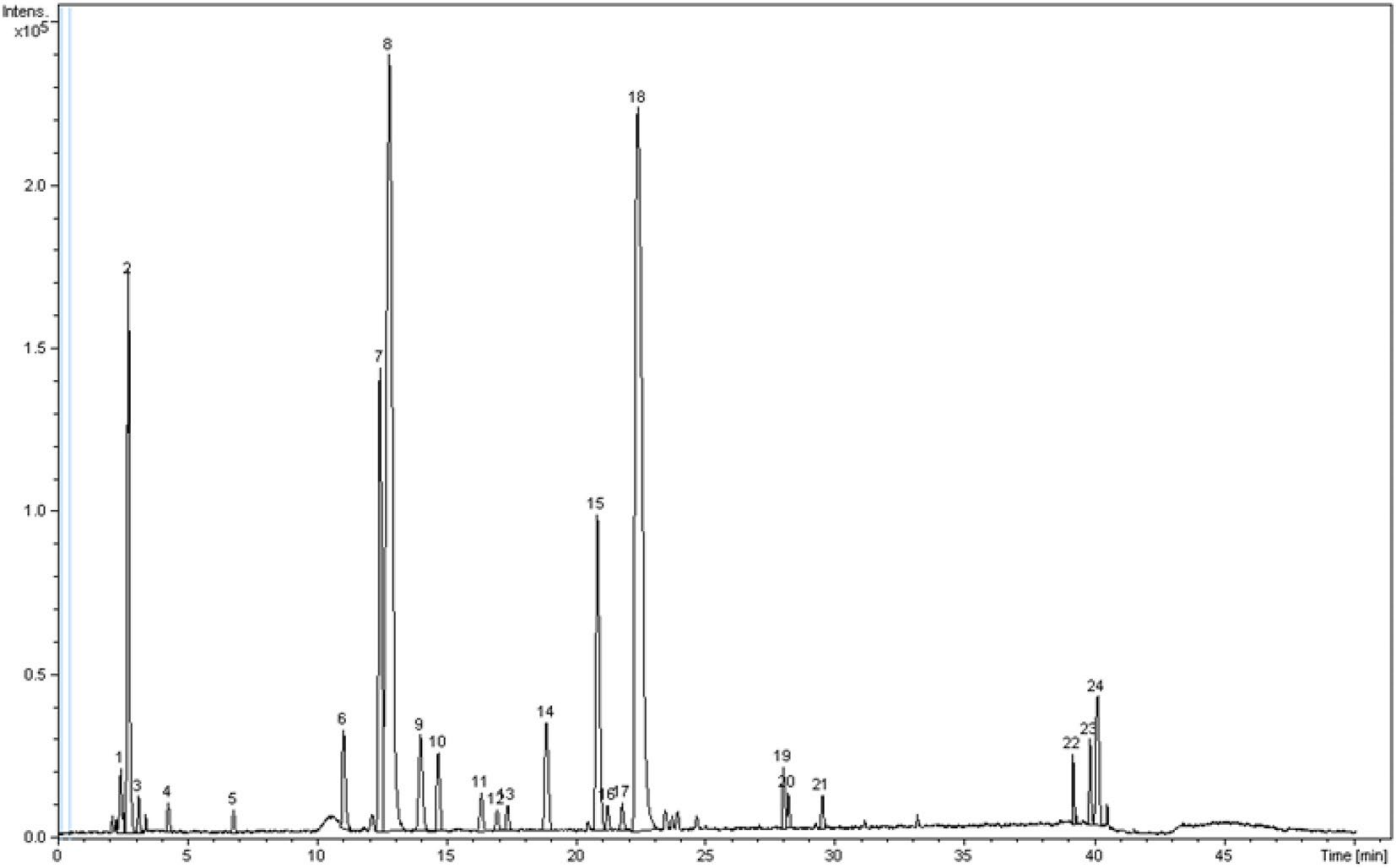
Liquid chromatography-mass spectrometry spectra of phenolic compounds in rambutan peel crude extract obtained from accelerated aqueous ethanol extraction under optimal conditions.

A total of 24 compounds were identified in rambutan peel extract, as shown in Fig. 5, with the main compounds based on m/z values^36–39^ shown in Table 6. Results concurred with Thitilertdecha et al.^40^, Hernández et al.^41^, and Méndez-Flores et al.^42^ who reported that the main phenolic compounds in rambutan peel extract were corilagin, geraniin and ellagic acid showing as peaks 7, 8 and 18 in Fig. 5, respectively. These compounds are found in the group of ellagitannins and recognized for their antioxidant activity. Thitilertdecha et al.^40^ reported that the total amount of three main compounds was 693.4 mg/g extract, as 53.5 mg of ellagic acid, 71.9 mg of corilagin and 568.0 mg of geraniin in rambutan peel extract. Peak 2 showed m/z of 173.05 with predicted molecular formula of C_7_H_10_O_5_ and was interpreted as shikimic acid that was also found in the extract. Yongliang et al.^36^ found shikimic acid at 7.39 mg/g in crude extract and at 0.18 mg/g in purified rambutan peel extract. Shikimic acid is an intermediate compound produced by the synthesis pathway of lignin, amino acids, alkaloids, phenolics, and phenylpropanoids of plants and microorganisms^43,44^, with use as an important material in the pharmaceutical industry. Saponin was also found in peel extract at peak 24 with retention time of 40.1 min and m/z 986.50. The predicted molecular formula of this compound is C_48_H_76_O_21_ and interpreted as medicagenic acid^39^. Please noted that the appropriate standards may be applied to confirm and quantify the contents of target phenolic compounds if further isolation and purification of the crude extract are needed.

**Table 6:** Major phenolic compounds in rambutan peel crude extract.

Tentative compounds	Retentiontime (min)	Formula	Group	Observed[M-H]^-^(m/z)	Theoretical[M-H]^-^(m/z)	References
Shikimic acid	2.7	C_7_H_10_O_5_	Cyclohexane carboxylic acid	173.0453	174.1513	Yongliang et al.^36^
Corilagin	12.4	C_27_H_24_O_18_	Ellagitannin	633.0753	634.4528	Fecka & Cisowski^37^
Geraniin	12.7	C_41_H_28_O_27_	Ellagitannin	951.0799	952.6452	Fecka & Cisowski^37^
Ellagic acid pentoside	20.8	C_19_H_14_O_12_	Ellagitannin	433.0426	433.0777	Oszmiański et al.^38^
Ellagic acid	22.3	C_14_H_6_O_8_	Ellagitannin	301.0003	300.9999	Oszmiański et al.^38^
Hex-Hex-Hex-Medicagenic acid	40.1	C_48_H_76_O_21_	Saponin	986.4989	987.4852	Pollier et al.^39^

### Inhibition of lipid oxidation in linoleic acid system

**Figure 6:**
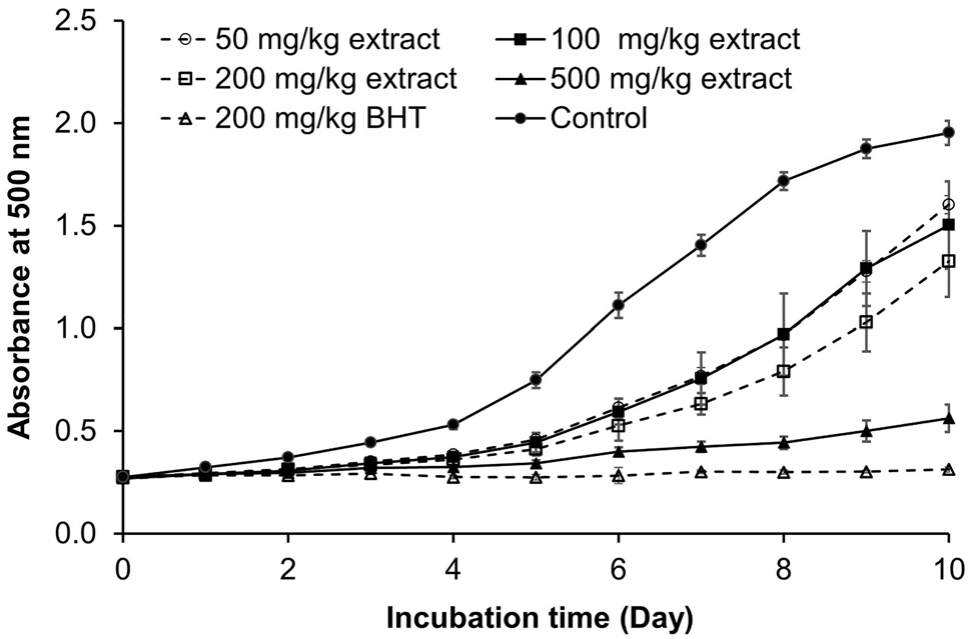
Absorbance at 500 nm of linoleic acid oxidation system containing various concentrations of rambutan peel crude extract (50, 100, 200 and 500 mg/kg) and 200 mg/kg of synthetic antioxidant (BHT).

The antioxidant activity of the sample was determined by the ferric thiocyanate (FTC) method, which monitors the inhibition of linoleic acid oxidation during 10 days. Oxidation of ferrous iron to ferric iron by lipid peroxides produces a colored complex when it reacts with ammonium thiocyanate. At 500 nm, a high absorbance implies a high quantity of linoleic acid oxidation products, whereas a low absorbance suggests a high level of antioxidant activity^23,24^. The extract sample showed lower lipid oxidation compared with the control treatment throughout incubation, while high concentration of extract effectively inhibited oxidation (Fig. 6). Extract samples were comparable to BHT (200 mg/kg) at the early stage of incubation (day 3) but only 500 mg/kg extract showed similar effectiveness to BHT after that. As a result, rambutan peel crude extract was less effective than BHT in inhibiting lipid oxidation at the same concentration but was still able to slow down lipid oxidation. Purification of this crude extract might improve its antioxidant capacity close to the synthetic compounds.

## Conclusions

Accelerated aqueous ethanol extraction was performed to extract polyphenol-rich crude extract from rambutan peel. This method was successfully optimized and validated using Box-Behnken design in combination with response surface methodology. Extraction temperature of 60 °C, extraction time of 34 min and ethanol concentration of 54 vol% provided maximum total phenolic and total anthocyanin contents, with highest ABTS scavenging activity and satisfactory extraction yield. Geraniin, ellagic acid, shikimic acid and corilagin were identified as the important active compounds found in this extract. Rambutan peel crude extract retarded lipid oxidation even at low concentration (50 mg/kg) and showed promise as a source of compounds with potential applications as natural antioxidants. The application of rambutan peel extract in real food systems is worthy for further study.

## Data Availability

All data generated or analyzed during this study are included in this published article.

## References

[1] Solis-Fuentes JA, Camey-Ortiz G, Hernandez-Medel Mdel R, Perez-Mendoza F, Duran-de-Bazua C (2010). Composition, phase behavior and thermal stability of natural edible fat from rambutan (*Nephelium lappaceum* L.) seed. Bioresour. Technol..

[2] Thitilertdecha N, Teerawutgulrag A, Rakariyatham N (2008). Antioxidant and antibacterial activities of *Nephelium lappaceum* L. extracts. LWT-Food Sci. Technol..

[3] Palanisamy UD, Ling L, Manaharan T, Appleton D (2011). Rapid isolation of geraniin from *Nephelium lappaceum* rind waste and its anti-hyperglycemic activity. Food Chem..

[4] Muhtadi M, Haryoto H, Sujono T, Suhendi A (2016). Antidiabetic and antihypercholesterolemia activities of rambutan (*Nephelium lappaceum* L.) and durian (*Durio zibethinus* Murr.) fruit peel extracts. J. Appl. Pharm. Sci..

[5] Li Y, Li Z, Hou H, Zhuang Y, Sun L (2018). Metal chelating, inhibitory DNA damage, and anti-inflammatory activities of phenolics from rambutan (*Nephelium lappaceum*) peel and the quantifications of geraniin and corilagin. Molecules.

[6] Zhuang Y, Ma Q, Guo Y, Sun L (2017). Protective effects of rambutan (*Nephelium lappaceum*) peel phenolics on H_2_O_2_-induced oxidative damages in HepG_2_ cells and d-galactose-induced aging mice. Food Chem. Toxicol..

[7] Mota MD, da Boa Morte AN, Silva LCRCE, Chinalia FA (2020). Sunscreen protection factor enhancement through supplementation with Rambutan (*Nephelium lappaceum* L) ethanolic extract. J. Photochem. Photobiol. B Biol..

[8] Phuong NNM, Le TT, Nguyen MVT, Camp JV, Raes K (2020). Antioxidant activity of rambutan (*Nephelium lappaceum* L.) peel extract in soybean oil during storage and deep frying. Eur. J. Lipid Sci. Technol..

[9] Li Y, Zhuang Y, Tian W, Sun L (2020). *In vivo* acute and subacute toxicities of phenolic extract from rambutan (*Nephelium lappaceum*) peels by oral administration. Food Chem..

[10] Mistriyani M, Riyanto S, Windarsih A, Rohman A (2021). Antioxidant activities and identification of an active compound from rambutan (*Nephelium lappaceum* L.) peel. Indones. J. Chem..

[11] Rajha HN (2014). Extraction of total phenolic compounds, flavonoids, anthocyanins and tannins from grape byproducts by response surface methodology. Influence of solid-liquid ratio, particle size, time, temperature, and solvent mixtures on the optimization process. Food. Nutr. Sci..

[12] Mikucka W, Zielinska M, Bulkowska K, Witonska I (2022). Recovery of polyphenols from distillery stillage by microwave-assisted, ultrasound-assisted and conventional solid–liquid extraction. Sci. Rep..

[13] Maran JP, Manikandan S, Nivetha CV, Dinesh R (2017). Ultrasound assisted extraction of bioactive compounds from *Nephelium lappaceum* L. fruit peel using central composite face centered response surface design. Arab. J. Chem..

[14] Dahmoune F, Nayak B, Moussi K, Remini H, Madani K (2015). Optimization of microwave-assisted extraction of polyphenols from *Myrtus communis* L. leaves. Food Chem..

[15] Teles ASC (2021). Combination of enzyme-assisted extraction and high hydrostatic pressure for phenolic compounds recovery from grape pomace. J. Food Eng..

[16] He L (2012). Subcritical water extraction of phenolic compounds from pomegranate (*Punica granatum* L.) seed residues and investigation into their antioxidant activities with HPLC–ABTS+ assay. Food Bioprod. Process..

[17] Herrero M, Martín-Álvarez PJ, Senorans FJ, Cifuentes A, Ibáñez E (2005). Optimization of accelerated solvent extraction of antioxidants from *Spirulina platensis* microalga. Food Chem..

[18] Gomes SVF (2017). Accelerated solvent extraction of phenolic compounds exploiting a Box-Behnken design and quantification of five flavonoids by HPLC-DAD in Passiflora species. Microchem. J..

[19] Figueroa JG, Borrás-Linares I, Lozano-Sánchez J, Quirantes-Piné R, Segura-Carretero A (2018). Optimization of drying process and pressurized liquid extraction for recovery of bioactive compounds from avocado peel by-product. Electrophoresis.

[20] García P (2021). Recovery of bioactive compounds from pomegranate (*Punica granatum* L.) peel using pressurized liquid extraction. Foods..

[21] Wolfe K, Wu X, Liu RH (2003). Antioxidant activity of apple peels. J. Agric. Food Chem..

[22] Truong VD, Hu Z, Thompson RL, Yencho GC, Pecota KV (2012). Pressurized liquid extraction and quantification of anthocyanins in purple-fleshed sweet potato genotypes. J. Food Compos. Anal..

[23] Rattaya S, Benjakul S, Prodpran T (2015). Extraction, antioxidative, and antimicrobial activities of brown seaweed extracts, *Turbinaria ornata* and *Sargassum polycystum*, grown in Thailand. Int. Aquat. Res..

[24] Mulyani S, Harsojuwono BA (2019). Relationship of turmeric and tamarind leaf extract ratio with induction time and antioxidant activity synergism. J. Appl. Hortic..

[25] Noor MHM, Lee KJ, Ngadi N (2021). Starch engineered with *Moringa oleifera* seeds protein crosslinked Fe_3_O_4_: A synthesis and flocculation studies. Int. J. Biol. Macromol..

[26] Samuagam L, Sia C, Akowuah G, Okechukwu P, Yim H (2013). The effect of extraction conditions on total phenolic content and free radical scavenging capacity of selected tropical fruits’ peel. J. Environ. Health..

[27] Yunusa AK (2018). DPPH radical scavenging activity and total phenolic content of rambutan (*Nephelium lappaceum*) peel and seed. Ann. Food Sci. Technol..

[28] Palanisamy U (2008). Rind of the rambutan, *Nephelium lappaceum*, a potential source of natural antioxidants. Food Chem..

[29] Ling LT, Radhakrishnan AK, Subramaniam T, Cheng HM, Palanisamy UD (2010). Assessment of antioxidant capacity and cytotoxicity of selected malaysian plants. Molecules.

[30] Khonkarn R, Okonogi S, Ampasavate C, Anuchapreeda S (2010). Investigation of fruit peel extracts as sources for compounds with antioxidant and antiproliferative activities against human cell lines. Food. Chem. Toxicol..

[31] Hippolyte MT, Augustin M, Hervé TM, Ndjouenkeu R, Somashekar D (2018). Application of response surface methodology to improve the production of antimicrobial biosurfactants by *Lactobacillus paracasei* subsp. tolerans N2 using sugar cane molasses as substrate. Bioresour. Bioprocess..

[32] Chew KK (2011). Effect of ethanol concentration, extraction time and extraction temperature on the recovery of phenolic compounds and antioxidant capacity of *Centella asiatica* extracts. Int. Food Res. J..

[33] Casazza AA, Aliakbarian B, Sannita E, Perego P (2012). High-pressure high-temperature extraction of phenolic compounds from grape skins. Int. J. Food Sci..

[34] Iglesias-Carres L (2019). Optimization and characterization of Royal Dawn cherry (*Prunus avium*) phenolics extraction. Sci. Rep..

[35] Kraboun K (2019). Influence of ethanol concentration on the extraction of monacolin K, γ-amino butyric acid (GABA) and antioxidant activity from angkak produced from germinated brown rice as a substrate. Food Appl. Biosci. J..

[36] Yongliang Z, Qingyu M, Yan G, Liping S (2017). Purification and identification of rambutan (*Nephelium lappaceum*) peel phenolics with evaluation of antioxidant and antiglycation activities in vitro. Int. J. Food Sci..

[37] Fecka I, Cisowski W (2005). Tannins and flavonoids from the *Erodium cicutarium* herb. Z. Naturforsch. B..

[38] Oszmiański J (2015). Analysis of phenolic compounds and antioxidant activity in wild blackberry fruits. Int. J. Mol. Sci..

[39] Pollier J, Morreel K, Geelen D, Goossens A (2011). Metabolite profiling of triterpene saponins in medicago truncatula hairy roots by liquid chromatography fourier transform ion cyclotron resonance mass spectrometry. J. Nat. Prod..

[40] Thitilertdecha N, Teerawutgulrag A, Kilburn JD, Rakariyatham N (2010). Identification of major phenolic compounds from *Nephelium lappaceum* L. and their antioxidant activities. Molecules.

[41] Hernandez C (2017). Polyphenolic content, in vitro antioxidant activity and chemical composition of extract from *Nephelium lappaceum* L. (Mexican rambutan) husk. Asian Pac. J. Trop. Med..

[42] Mendez-Flores A (2018). Ultrasound-assisted extraction of antioxidant polyphenolic compounds from *Nephelium lappaceum* L. (Mexican variety) husk. Asian Pac. J. Trop. Med..

[43] Bochkov DV, Sysolyatin SV, Kalashnikov AI, Surmacheva IA (2012). Shikimic acid: Review of its analytical, isolation, and purification techniques from plant and microbial sources. J. Chem. Biol..

[44] Rawat G, Tripathi P, Jahan F, Saxena RK (2013). A natural isolate producing shikimic acid: Isolation, identification, and culture condition optimization. Appl. Biochem. Biotechnol..

